# Causal interhemispheric neuromodulation sharpens synaptic and neurobehavioral inhibition in stroke

**DOI:** 10.1093/braincomms/fcag119

**Published:** 2026-04-13

**Authors:** João Castelhano, Felix Duecker, Ana Carolina Xavier, Nádia Canário, Isabel C Duarte, Sónia Afonso, Ana Gabriel Marques, Cecilia Lourenço, Filipe Carvalho, Jorge Lains, Angela Neves, João Sargento Freitas, Gustavo Cordeiro Santo, Antero J Abrunhosa, Alexander T Sack, Miguel Castelo-Branco

**Affiliations:** CIBIT/ICNAS University of Coimbra, 3000-548 Coimbra, Portugal; Department of Cognitive Neuroscience, Faculty of Psychology and Neuroscience, Maastricht University, Oxfordlaan 55, 6229 EV Maastricht, the Netherlands; CIBIT/ICNAS University of Coimbra, 3000-548 Coimbra, Portugal; CIBIT/ICNAS University of Coimbra, 3000-548 Coimbra, Portugal; CIBIT/ICNAS University of Coimbra, 3000-548 Coimbra, Portugal; CIBIT/ICNAS University of Coimbra, 3000-548 Coimbra, Portugal; Centro de Medicina de Reabilitação da Região Centro—Rovisco Pais, 3060-908 Tocha, Portugal; Centro de Medicina de Reabilitação da Região Centro—Rovisco Pais, 3060-908 Tocha, Portugal; Centro de Medicina de Reabilitação da Região Centro—Rovisco Pais, 3060-908 Tocha, Portugal; Centro de Medicina de Reabilitação da Região Centro—Rovisco Pais, 3060-908 Tocha, Portugal; CIBIT/ICNAS University of Coimbra, 3000-548 Coimbra, Portugal; Stroke Unit, ULS-CHUC, 3000-075 Coimbra, Portugal; Stroke Unit, ULS-CHUC, 3000-075 Coimbra, Portugal; CIBIT/ICNAS University of Coimbra, 3000-548 Coimbra, Portugal; Department of Cognitive Neuroscience, Faculty of Psychology and Neuroscience, Maastricht University, Oxfordlaan 55, 6229 EV Maastricht, the Netherlands; CIBIT/ICNAS University of Coimbra, 3000-548 Coimbra, Portugal; Department of Cognitive Neuroscience, Faculty of Psychology and Neuroscience, Maastricht University, Oxfordlaan 55, 6229 EV Maastricht, the Netherlands

**Keywords:** neurostimulation, transcranial magnetic stimulation (TMS), multimodal neuroimaging, molecular imaging, brain plasticity

## Abstract

Hemispheric functional asymmetries are a key aspect of human brain organization. Revealing how causal manipulations of hemispheric (im)balances dynamically change pre- and post-synaptic GABA regulation, in terms of mean and variance changes, is essential to understand the plasticity of brain asymmetries in health and disease. This has direct implications for treatment approaches in stroke relying on interhemispheric inhibition using transcranial magnetic stimulation. We investigated neurobehavioral and neurochemical effects of cortical stimulation using a unique multimodal brain imaging and stimulation set-up, in both healthy and unilaterally lesioned hemispheres, in stroke. We performed two molecular imaging sessions measuring GABA receptor levels, with sham and real neurostimulation, functional magnetic resonance imaging (MRI) and GABA neurospectroscopy, all in the same day. We found that noninvasive inhibitory neuromodulation of interhemispheric interactions causes pre- and post-synaptic sharpening of GABA neurotransmitter and receptor levels, providing a mechanism of action for transcranial magnetic stimulation contralateral inhibitory protocols in stroke. Moreover, we found a positive association between GABA neurotransmitter and receptor levels, suggesting increased synaptic coupling. This discovered plasticity mechanism, based on correlated reduction of pre- and post-synaptic variability, represents a basic principle underlying long-range inhibitory hemispheric interactions and allows for the stabilization of synapses and rebalancing of brain activity in disorders of hemispheric imbalance, such as stroke.

## Teaser

Castelhano *et al.* report that inhibitory neuromodulation over the parietal cortex in stroke patients led to increased homogeneity of GABA and receptor binding levels, along with their augmented positive association, suggesting increased synaptic coupling as a molecular mechanism of neuromodulation. They found a recovery of brain activation contralateral to the stimulation.

## Graphical Abstract

**Graphical Abstract:**
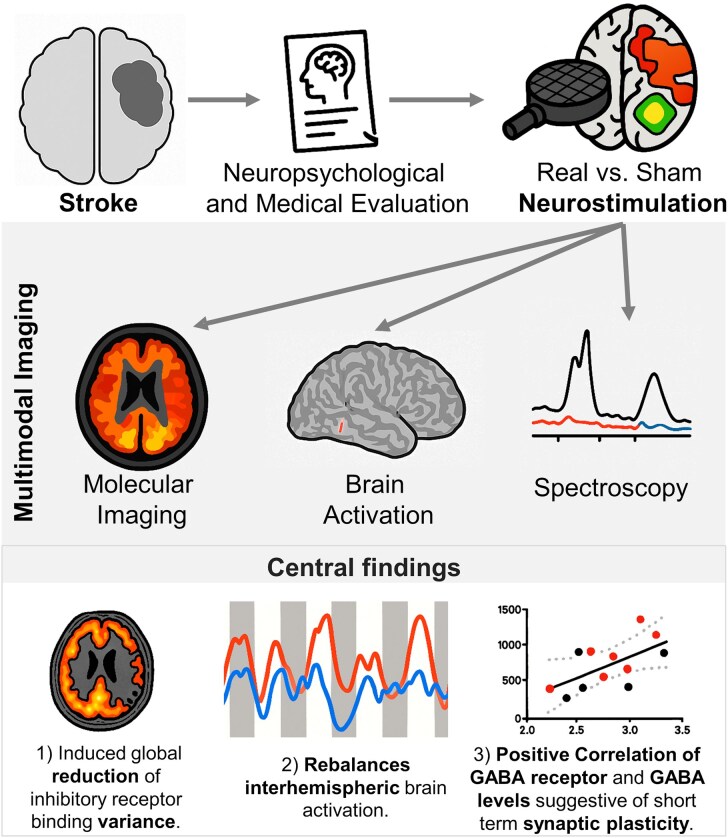

## Introduction

Functional differences between the left and right hemispheres are present in many species, and there are countless examples of behavioural manifestations of this lateralization principle of brain organization.^1,2^ Although hemispheric lateralization has been established across multiple cognitive domains, the underlying nature of hemispheric interactions and resulting asymmetries remains poorly understood. In particular, the relation to synaptic mechanisms is unknown, and a reduction in basic biological principles is therefore lacking. The biological principles underlying these hemispheric asymmetries and their relation to synaptic mechanisms, however, remain unknown. Unravelling how interhemispheric organizational principles are related to the molecular regulation of synaptic function is instrumental in understanding one of the most basic principles of human brain organization, as well as functional deficits related to acquired hemispheric imbalances.

TMS can be used to experimentally modulate hemispheric interactions and induce lasting neuroplastic changes in hemispheric asymmetries. However, while established on behavioural and neural network levels, we still lack direct evidence by which exact biological and molecular mechanisms these effects are caused, and hence by which neurobiological principles these general hemispheric functional asymmetries are organized.^3^

Cellular and computational studies have shown that synaptic parameters can be derived not solely from mean values, but that variance and covariance in neural measures are a rich source of information.^4^ Accordingly, variance analysis can be used as a tool to predict the mechanism underlying synaptic plasticity.^5^ Importantly, modelling work has suggested that variability of postsynaptic responses depends nonlinearly on the number of synaptic inputs,^6^ which may be changed by transcranial stimulation. Whether these principles apply to humans remains an open question. A recent study suggested that this might be the case, by showing that increased variability of excitatory drive to fast-spiking parvalbumin interneurons, as suggested by postmortem molecular studies in schizophrenia, might lead to lower prefrontal gamma power, as further suggested by a computational model.^7^

In this line, we hypothesize that synaptic variability plays a critical role in hemispheric asymmetries, where postsynaptic response optimization at inhibitory synapses might lead to the desired optimal excitation/inhibition balance between hemispheres through variance reduction, in line with prior modelling and cellular work.^8^ Long-term modifications of neuronal connections are critical for learning and plasticity. Their locus of expression—pre- or post-synaptic—and respective covariance remains debated, which poses challenges to their measurement using positron emission tomography (PET) or spectroscopy.^9^ In line with the above-mentioned theoretical framework, short and long-term plasticity may depend on optimization of the postsynaptic response statistics toward a given mean with minimal variance. The state of the synapse may depend on the ratio of pre- and post-synaptic modifications. Such changes in pre- and post-synaptic variability may also be the driving force underlying various forms of neuroplasticity.^10^ Approaches to rebalance the hemispheres to alleviate functional deficits resulting from dysfunctional hemispheric interactions provide a way to infer on biological significance. Conceptual models and a combination of causal approaches, based on neurostimulation and/or lesion studies, are instrumental in this respect. Combining TMS with unilateral lesion studies (e.g. unilateral stroke models) provides a unique opportunity to test specific theories of interhemispheric interaction^11,12,13^ and their molecular basis. We also have functional MRI (fMRI) evidence for the interhemispheric inhibition model.^14,15^ Our previous studies in the motor cortex of stroke patients show that continuous theta burst stimulation (cTBS) can be used as an inhibitory protocol for the unaffected hemisphere.^16-18^ Although cTBS is classically described as inhibitory, its network-level effects might depend on the state of the system being stimulated (e.g. lesioned versus intact, hyper- versus hypo-excitable).^19,20^ This has direct implications for treatment approaches of stroke relying on interhemispheric inhibition using TMS.

The interhemispheric inhibition model postulates that both hemispheres are directly interconnected and mutually controlling in a domain-specific manner, that is, inhibiting each other within an equilibrium of an optimally tuned excitation/inhibition balance. After unilateral brain damage, the competition between hemispheres is highly biased, leading to decreased activity in the lesioned hemisphere but also increased activity in the contralesional hemisphere due to disinhibition.^21,22^ The result is an imbalance between hemispheres with large functional consequences.^12^ In this context, lateralized deficits are not merely due to a loss of function of the lesioned hemisphere but may also be the result of disrupted interactions between hemispheres at a synaptic level, further amplifying and consolidating these deficits. This provides a perfect model to experimentally study dynamic processes of hemispheric lateralization and functional asymmetries, and how the changes in synaptic variability can be implicated in the process. While the interhemispheric inhibition model has traditionally informed noninvasive brain stimulation approaches, recent perspectives—such as the bimodal balance–recovery model^23^—highlight the need for individualized, network-based interpretations. These and other studies, including the critique articulated by Carson,^24^ have emphasized that callosal projections do not generate generalized or undifferentiated inhibition, but instead, exert contrast-enhancing, region-specific influences through mechanisms such as crossed surround inhibition and, in some contexts, crossed facilitation. Facilitatory connections with the homotopic area of the opposite motor cortex are postulated, being surrounded by a more extensive zone in which inhibitory responses to transcallosal stimulation are obtained. These mechanisms narrow the excitatory focus in a highly differentiated way, underscoring that the classical interhemispheric inhibition model may not universally explain stroke-recovery dynamics across all stages, lesion types, or behavioural domains. Such alternative frameworks may be relevant beyond motor recovery.

TMS has been proposed as a technique that can be used to selectively modify brain hemispheric (im)balance.^11,12^ It provides a noninvasive, reversible, and relatively localized approach that has substantial promise for particular clinical applications,^25,26^ including stroke rehabilitation.^27^ However, the effects of TMS and the link between molecular, neurochemical, physiological, and behavioural responses^21,22^ are still enigmatic, in particular, in the context of interhemispheric interactions. Here, we uniquely combined multimodal and dual causal (TMS/lesion) strategies to address these questions. Unilateral stroke is a condition that provides a unique opportunity to test the hemispheric (im)balance hypothesis because it allows for the direct assessment of the interaction between a healthy and lesioned hemisphere within the same participant. Furthermore, it is important to tailor and optimize interventions in stroke patients as well as to investigate possible ways to modulate GABA poststroke. To achieve this goal, understanding the cellular and network mechanisms underlying the plasticity in stroke is instrumental, and previous studies suggested that changes in GABAergic neurotransmission play a particularly important role.^9^ Such a combination of approaches allowing for deep physiological dissection is hard to achieve in healthy individuals.^17^ The current approach aims at understanding the underlying imbalance at a molecular level, following the above-mentioned synaptic plasticity hypothesis, which is pivotal to understanding the principles of human brain organization underlying lateralization and implementing novel model-based treatment approaches in disorders such as stroke.^28^

## Materials and methods

### Study experimental design

**Figure 1:**
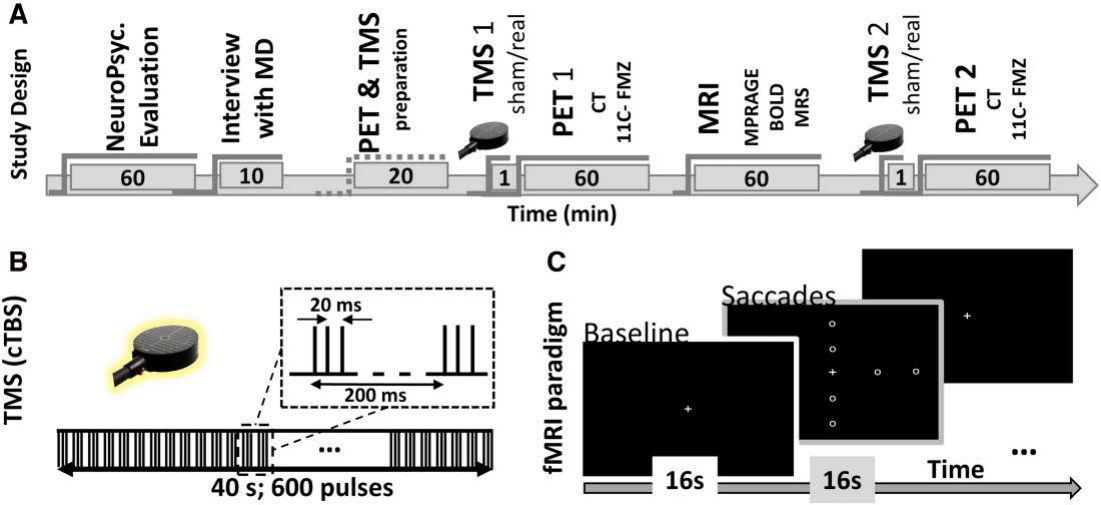
**Study design, procedures.** (**A**) In this study, we tested unilateral stroke patients (N = 19) during a TMS intervention. Subjects performed a full day of tests that started with a neuropsychological evaluation, followed by an interview with the medical doctor (MD) to evaluate the general health of the subject to perform the imaging study. Imaging study included a first test with TMS to define each individual resting motor threshold (rMT), followed by two TMS stimulations (sham/active; single-blinded), two positron emission tomography (PET) flumazenil (11C-FMZ) with CT acquisitions and one magnetic resonance imaging (MRI) session with structural (MPRAGE, magnetization prepared rapid gradient echo)), functional (blood oxygenation level dependent, BOLD) and spectroscopy (MRS) acquisitions. (**B**) A continuous theta burst protocol (cTBS) was applied, that is, 50 Hz triplets at a 5 Hz for 40 s (= 600 pulses). (**C**) Participants performed a simple eye movement attentional fMRI paradigm; alternating 10 blocks of 16 s of baseline (fixation cross) and 10 blocks of Saccades (gaze to a target in 1/8 positions in the screen).

We combined TMS, MRI and PET to experimentally manipulate the (im)balance between hemispheres. The experiment included a full day of testing with the following acquisitions (Fig. 1 Timeline): patients arrived from the rehabilitation hospital to our institute in the morning and started a full neuropsychological evaluation, followed by a medical interview to evaluate the conditions to proceed with the exams. Next, they were prepared for PET imaging and performed a short assessment of resting motor threshold (rMT) with TMS. Then, a first TMS (real or sham) was applied, followed by a PET- 11C- Flumazenil imaging acquisition and one MRI acquisition (including anatomical, functional and spectroscopy recordings). After MRI, patients rest for about 1 hour, and a second TMS (real or sham) was applied immediately before the second PET imaging. Although this was an extensive protocol, all patients were very collaborative and able to perform the experiment. Further technical details on the procedures are described in the subsections below.

### Participants

Patients inclusion criteria were based on the following scores from the National Institutes of Health Stroke Scale: presence of extinction and inattention (item 11 > 0); first screening for the presence of neglect; absence of hemianopia (item 3 = 0, unless in case of extinction) to exclude perceptual deficits; absence of severe aphasia (item 9 ≤ 1) to ensure sufficient communication abilities; and sufficient strength in right arm (item 5 ≤ 1) to ensure that patients can do tasks with their right hand.

Nineteen stroke patients (9 females; mean age = 66.71 years; detailed clinical characteristics of the patient cohort—including lesion volume, stroke severity, and impairment measures—are provided in Table 1), right-handed and with an ischemic lesion in the right hemisphere of the brain, were informed of the objectives and potential risks of the study and signed a written consent inform. The study was approved by the Ethics Committee of the Faculty of Medicine of the University of Coimbra and was conducted in accordance with the Declaration of Helsinki.

**Table 1:** The demographic details of our sample

	Total	TMS Real (N)	TMS Sham (N)
N	19	16	17
Males	10	7	9
Age (years), mean ± SD	66 ± 8.1		
Age (years), range	51–87		
Time since stroke, mean (range in weeks)	5 (1–8)		
PET Act. Adm. (mCi) mean ± SD	15.5 ± 0.46	16	17
MRS	16	7	9
fMRI	13	7	6
Landmark Test mean ± SD	0.93 ± 0.08		
Token Test total score mean ± SD	20.63 ± 1.83		
MoCA total score mean ± SD	23.53 ± 4.91		
Lesion Location	Fig. 2		
Lesion Hemisphere (left/right)	0/19		
Total Lesion Volume mm3	135824		
Group Lesion Centroid (MNI: X,Y,Z)	34, −6, 27		

The number of subjects per condition and the average group scores for the neuropsychological tests are also reported (values are matched with the normative values for the general population; SD = standard deviation)

### Neuropsychological evaluation

Behavioural measures were acquired in a neuropsychological evaluation comprising the landmark test, token test, Montreal cognitive assessment (MoCA), and behavioural inattention test.

### Brain stimulation

TMS was delivered using a MagPro X100 magnetic stimulator connected to a round coil (MCF-125; MagVenture, Denmark). We target the contralesional hemisphere (left Intra-parietal sulcus (IPS)) with a stimulation protocol that is well-known to decrease cortical excitability.

The TMS protocol was single-blinded and as follows: (i) the coil was positioned over the contralesional primary motor cortex at a 45° angle to the sagittal plane. The motor hotspot was identified as the scalp location eliciting consistent first dorsal interosseous (FDI) muscle contractions. Individual resting motor thresholds (rMTs) were then determined using standard criteria (i.e. the lowest stimulation intensity that produced observable muscle twitch in at least 5 out of 10 trials). (ii) Determine stimulation site over parietal cortex: given the large experimental load for the participants, we relied on the international 10/20 electrode system to guide our coil positioning. Specifically, the round coil was positioned over P3 and oriented with the help of a template of the TMS coil at the correct location. To ensure successful brain stimulation in all our patients, we decided to establish a compromise by using a somewhat less focal round TMS coil, but ensuring more focality than most round coils. Traditional round coils have the strongest magnetic field at the outer rim. However, we used a round coil that is wired in a way resulting in a magnetic field that is strongest at the centre of the coil. (iii) TMS protocol^29^: a continuous theta burst protocol was applied, that is, 50 Hz triplets at a 5 Hz for 40 s (= 600 biphasic pulses; Fig. 1 B). This protocol is applied to the healthy hemisphere to test our hypothesis that reducing the activity of this hemisphere can increase the activation at the contralateral hemisphere (the right hemisphere in our case). The stimulation intensity was defined as 80% of the individual rMT (mean rMT = 45.97 ± 3.09%), which is associated with more consistent theta burst effects,^30^ and TMS studies in clinical populations tend to favour the use of the resting motor threshold when applying theta burst protocols, with actually less variable effects.^31,32^ Since the gap between TMS application and the PET scan should be as short as possible, the TMS protocol was performed while the subjects were already positioned in the PET scanner and initiated as late as possible, before the tracer was administered (9 min: 31 s on average). During stimulation, the coil was maintained at the target point using an adjustable coil holder. TMS real (real stimulation) and TMS sham (sham stimulation) were applied before each PET scan in a counterbalanced order. The sham was applied with exactly the same protocol, with the coil close to the patient’s head but oriented to the scanner bed instead of the participant’s head. This way, we ensured a ‘feeling of being stimulated’ without stimulation for the sham recordings. The patients were not able to distinguish between real and sham conditions (self-report at the chance level).

### Image acquisition

#### PET imaging

Patients receive TMS (sham and real stimulation; single-blinded) each one followed by a computed tomography (CT) scan and a three-dimensional PET of the entire brain (90 slices, 2-mm slice sampling; Philips Gemini GXL). We used PET 11C-flumazenil (FMZ) imaging to measure GABA-A receptor binding in all stroke patients. Flumazenil is a competitive antagonist of the GABA-A receptor, and it binds to the benzodiazepine site of the receptor. This binding site is largely and predominantly located in the postsynaptic compartment.^33^ Each dynamic list mode PET 11C-flumazenil scan was acquired over a period of 60 min, after each TMS session.

#### MRI

All patients performed 3T MRI (anatomical scan, fMRI with a simple saccade paradigm and spectroscopy). MRI data were acquired after both real and sham TMS sessions, depending on the condition used during the initial PET scan. Scanning was performed using a 3T Tim Trio (Siemens, Erlangen, Germany) with a 12-channel head coil for signal reception, approximately at the same time-of-day for all patients to overcome possible daily regulation of cortical excitation/inhibition balance effects.^34^ T1-weighted structural images of the brain were acquired with a Magnetization Prepared Rapid Gradient Echo Imaging (MPRAGE) sequence with 1 mm^3^ isotropic voxel, echo-time (TE) = 3.42 ms; repetition time (TR) = 2530 ms; slices = 176; field of view (FoV) = 256 mm; flip angle (FA) = 7.0; inversion time 1100 ms; 256 × 256 matrix; and GRAPPA acceleration factor = 2. fMRI data was acquired with the following parameters: repetition time TR = 2000ms; echo-time TE = 30 ms; slices = 37; flip angle FA = 90°; field of view FoV = 192 mm; slice thickness = 3 mm; number of volumes = 169. The fMRI run lasted 5 min 40 s.

**Figure 2:**
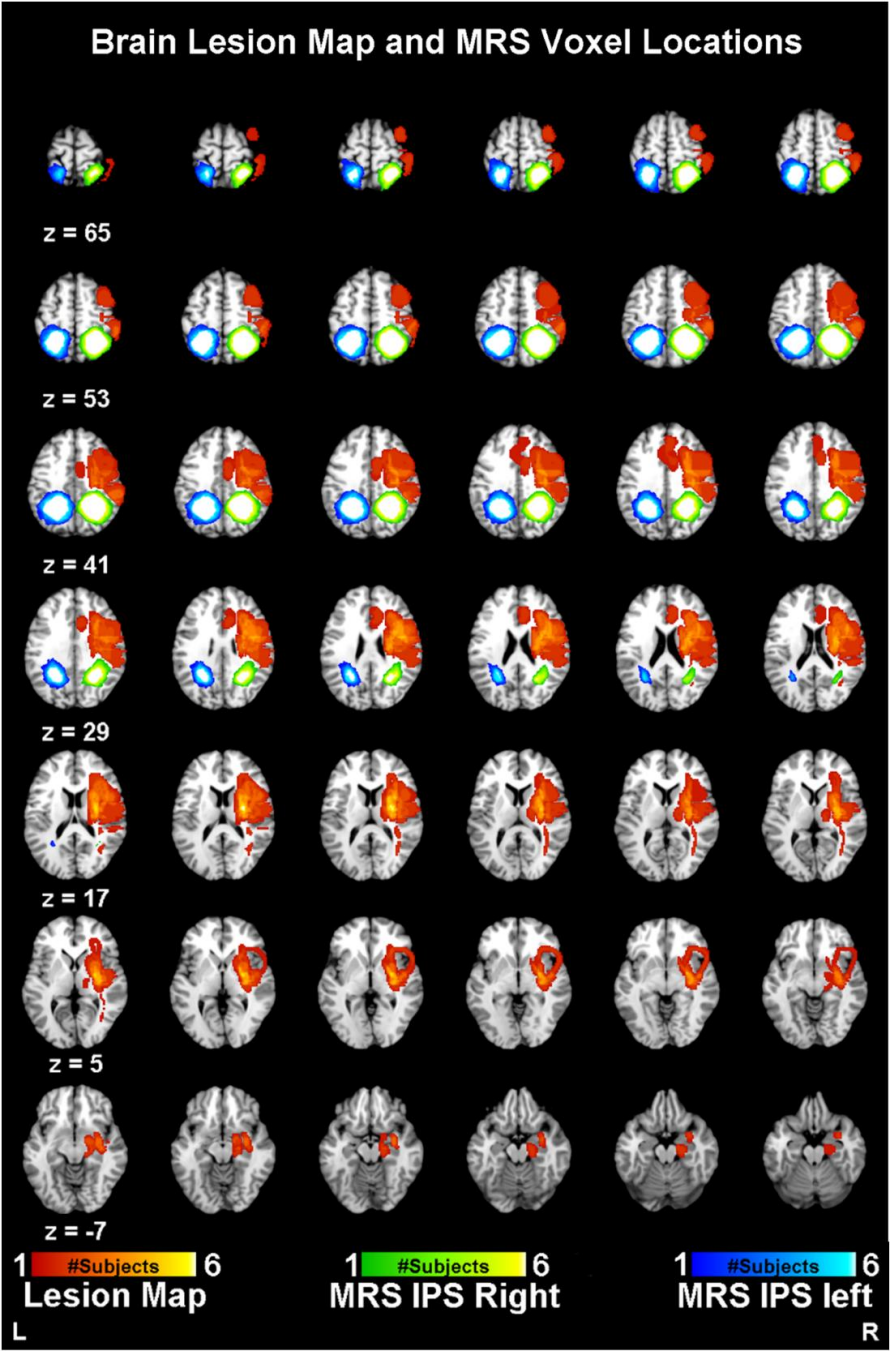
**Representation of the group lesion map and spectroscopy (MRS) voxel locations.** Lesions were segmented for each patient from the structural MRI data (orange map). Spectroscopy IPS voxels (Green—IPS right; Blue—IPS left) were extracted from Gannet software coregistration outputs. Group (lesion and MRS voxel; N = 13) colormaps represent the homogeneity, size and location across the patients. L—left; R- right.

Two H-MRS voxels were placed over the left and right IPS; Fig. 2). Individual voxel placements were based on anatomical landmarks and did not overlap with lesioned tissue. The group-averaged voxel location is shown in Fig. 2 alongside the lesion map, with minimal average overlap (2.09%) for visual comparison. We used the Hadamard Encoding and Reconstruction of MEGA-Edited Spectroscopy (HERMES) approach to quantify GABA and Glutamate + Glutamine (Glx). The sequence parameters were as follows: volume 30 × 30 × 30 mm; TR = 2000ms; TE = 80 ms; Averages = 392; FA = 90; water suppression bandwidth 50 Hz; delta frequency = -1.7 ppm; edit pulse 1 frequency =1.9 ppm; edit pulse 2 frequency =4.56 ppm; edit off frequency = 7.5 ppm. Two spectra with and without water suppression were recorded for further spectrum normalization. In our protocol, a brief interval (voxel positioning) was inserted between the fMRI and MRS acquisition to allow for signal stabilization. Both sets were collected for real and sham TMS sessions to allow for direct comparison against a baseline condition (the sham TMS).

#### fMRI task description

During functional acquisitions, the scanner room was dimmed, and participants were asked to perform a saccade each time a target appear in the screen. During the baseline blocks (11 blocks; 8 TRs of duration each), the subject had to fixate a cross in the centre of the screen. In the saccade blocks (10 blocks, 8 TRs each), a white dot appear in the screen for 500 ms, randomly presented at 8 distinct locations (2 up, 2 down, 2 left, 2 right), and subjects had to perform a saccade towards the target each time it appeared (Fig. 1 C). Saccades were purely driven by the information provided by the target dot showing up, thus strongly emphasizing voluntary control mechanisms and the voluntary execution of saccades. Stimuli were presented on a 40-inch liquid crystal display Nordic NeuroLab monitor at the back of the scanner, 1825 mm viewing distance. The video mode was 1920 × 1080 at 60 Hz, and the background luminance was 100 cd/m^2^. The Presentation software package (NeuroBehavioural Systems, Albany, CA, United States) was used to control stimulus presentation and synchronization with the fMRI acquisition. To confirm the compliance with the task, we recorded eye movement data (Eyelink 1000 plus, SRResearch, Canada) during the experiment at a sampling rate of 1 kHz.

### Image data processing and analysis

#### PET processing

**Figure 3:**
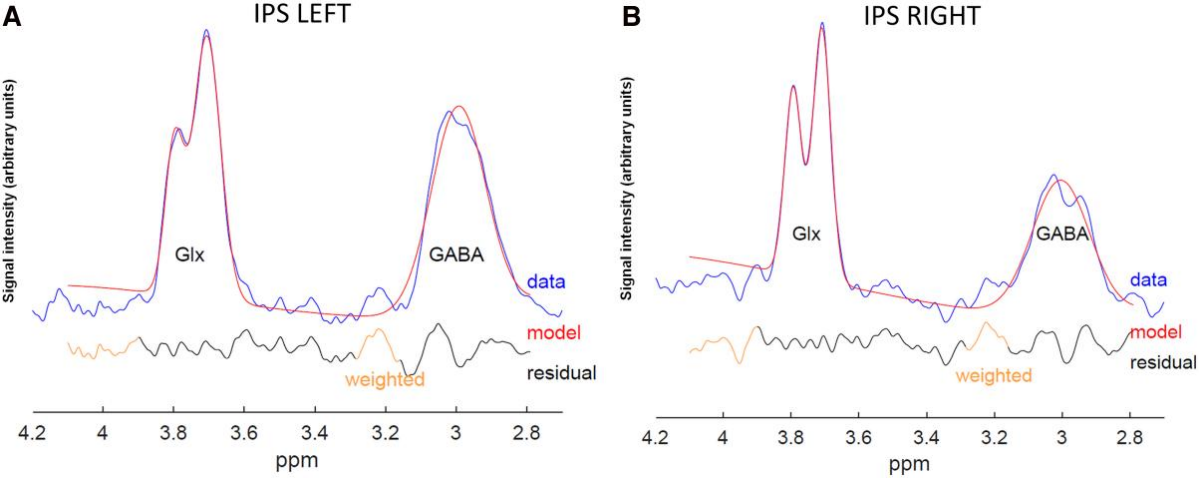
**Edited spectrum and Model fit individual example.** MRS data were acquired with HERMES sequence: the fit output for the Glx and GABA signals are shown on the plots for the left (**A**) and right (**B**) intra-parietal sulcus (IPS) acquired after TMS real. Individual spectra were processed using the Gannet 3.0 toolkit, the blue line shows the raw preprocessed data, the red line is the fit of the model, and the black line is the residual difference between the experimental data and the curve fit. The quality-control (QC) values for this IPS left example spectra are as follows: full-width half maximum (FWHM) 9.5 Hz, Cramér–Rao Lower Bounds (CRLB) for Glx = 4.6% and GABA = 5.4%. Individual QC IPS right: FWHM 8.7 Hz, CRLB Glx =5.1%, CRLB GABA = 7.3%.

PET data were reconstructed using a Line-of-Response Row-Action Maximum Likelihood Algorithm (LOR-RAMLA) algorithm. PET 11C-Flumazenil voxelwise binding potential (BP) was obtained using in-house made software (Fig. 3), with the modified reference tissue method 2 (MRTM2) as compartmental model and the pons as reference. PET and MRI data were normalized to Montreal Neurological Institute (MNI) space; the same geometric transformation was used after the PET scan had been rigidly coregistered with the correspondent anatomical MRI scan. We performed whole-brain and region-of-interest (ROI)-based analyses. ROI-based quantification was performed in regions used for spectroscopy (IPS—left and right) for both real and sham TMS sessions.

#### MRS processing

Quantification of MRS data was performed using the Gannet 3.0.^35^ GABA, Glx and glutathione, in addition to creatine signals, were obtained from the difference edited spectra. The peaks for each metabolite were fitted to a simple Gaussian model. Creatine signal was fitted to a double Lorentzian model. GABA signal is known to be contaminated by other macromolecules and tissue composition.^36^ To overcome this, we performed tissue correction^35^: to account for differences in voxel tissue composition, tissue-specific relaxation and water visibility values were taken into account, and the segmentation of voxel fraction of white matter (fWM) and grey matter (fGM) was used to normalize the GABA + concentration according to: GABA + corr = GABA+/(fWM + fGM). This full tissue normalization results in a GABA + value, which considers the average voxel tissue composition for both real and sham conditions. Moreover, water frequency drift and fit errors of the peaks were calculated to provide a measure of MRS data quality. In this line, both sham and real TMS spectral quality were assessed using signal-to-noise ratio (SNR) and Cramér–Rao Lower Bounds (CRLB), with metabolite estimates included only if CRLB was <10%. This threshold ensures reliable and high confidence in the metabolite quantification fitting results of GABA + and glutamate signals. Note that metabolite levels were reported in institutional units (I.U.^37^). An example of the spectrum and fit output is shown in Fig. 3.

#### Processing of structural images

We performed the preprocessing of structural MRI data using BrainVoyager Qx (Brain Innovation) with the default pipeline settings. The brain representations in the figure panels were also created in this software. Structural images were normalized to the AC-PC space. Raw image data are available from our institution’s repository upon request, and Fig. 2 shows the group lesion region map.

#### Processing of functional images

The functional images were preprocessed with the default pipeline settings. 3D body motion correction, aligning all subsequent functional runs to the closest one to the anatomical scans, and temporal high-pass filtering (general linear model GLM-Fourier with two cycles sine/cosine per run, including linear trend removal) were applied to the functional data. Furthermore, the preprocessed images were registered to the structural space. Neural responses to the saccade task were assessed by applying a GLM analysis.^38^ Predictors for each condition were created by convolving the stimulation blocks with a standard hemodynamic response function.^39^ The fixation blocks were considered as baseline. Data were normalized with z-transformation and corrected for serial correlations with a second-order autoregressive method.^40^ A repeated measures ANOVA (factors: task and stimulation) was used to test for the effect of TMS on brain activation. A group GLM analysis was used to reveal the neural network associated with the saccades task for both TMS real and sham conditions.

### Statistical analysis

Throughout the manuscript, and unless stated otherwise, significance for all fMRI results was assessed using cluster inference with a cluster-defining size of 300 voxels and a cluster probability of *P* < 0.05 FDR-corrected for multiple comparisons. Correlations were calculated with the Spearman correlation algorithm. The variances were calculated by taking the average of the squared differences of each data point from the sample's mean for each parameter (e.g. PET: using each voxel of the ROIs; GABA measures variance across subjects). The differences in the variances were tested with Levene’s test, and comparison between conditions was tested with Mann-Whitney at a significant threshold of 0.05 (FDR-corrected for multiple comparisons).

## Results

### TMS caused a reduction in GABA receptor binding variance

**Figure 4:**
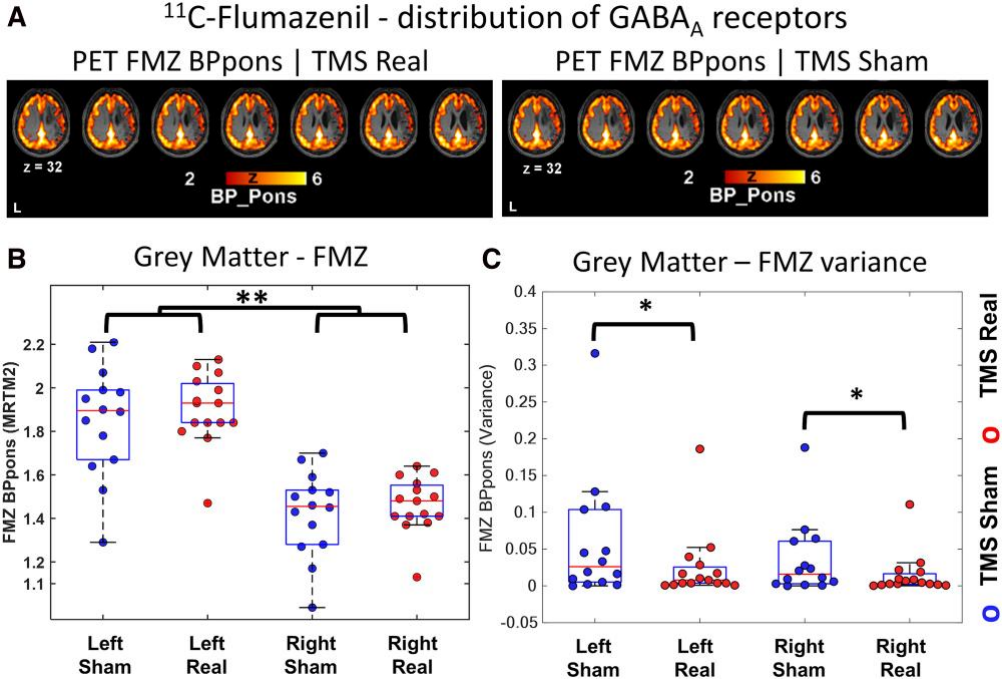
**PET 11C-FMZ shows TMS-induced global reduction of GABA receptor binding variance**. (**A**) Quantification of PET data reveals the distribution of GABA_A_ receptors in the brain. A group average binding potential (BP) map is shown for TMS Real and Sham. (**B**) FMZ quantification in left and right hemispheres grey matter (GM) revealed a significant reduction of GABA_A_ receptors in the right (lesioned) hemisphere GM (**P < 0.0001) and most importantly (**C**) a significant effect of the TMS in reducing the groups’ variance (LeveneF(26) = 4.62, * *P* = 0.041). Each individual datapoint represents a single patient (N = 16 real; N = 17 sham TMS).

We did the quantification of the whole-brain distribution of GABA receptors using the PET-FMZ (Fig. 4.A).^33^ We found a significant reduction (t(56) = 8.83, *P* < 0.0001, Cohen d = 2.32) of GABA_A_ receptor binding in the right (lesioned) hemisphere (Fig. 4.B). Importantly, we found significant differences in receptor level variance but not in their means between the TMS real and sham conditions: TMS caused a reduction in GABA receptor binding variance (Fig. 4.C; LeveneF(26) = 4.62, *P* = 0.041). The stimulation target region was the left IPS, and we performed ROI-based analyses (IPS left and right) of PET data. ROIs were generated for PET analyses based on the MRS voxels’ position (Fig. 2). PET-FMZ data did not show significant differences between left/right IPS neither TMS On versus Off in this region.

### TMS rebalances brain activation during a Saccadic task

**Figure 5:**
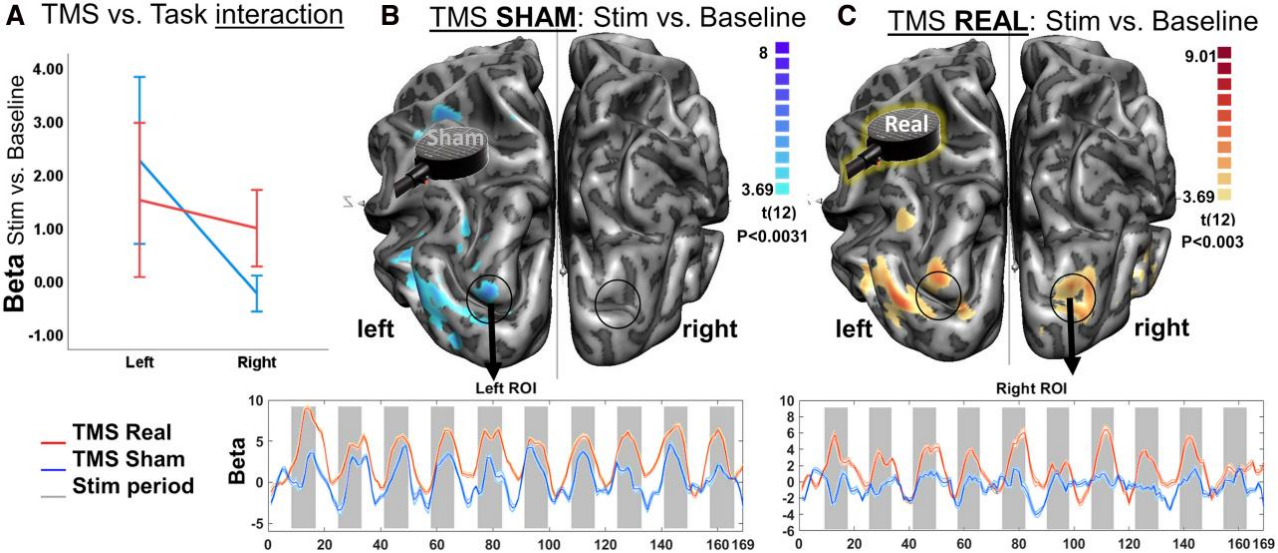
**TMS rebalances brain activation during a Saccadic task.** (**A**) Activation changes during the eye movements task: we found a significant interaction effect of the TMS stimulation versus saccade task for the left hemisphere (*P* < 0.0062; N = 13), corroborating the direct focal impact of TMS in left IPS. (**B**) TMS Sham: brain activation during the saccade task (stimuli > baseline) was increased in the oculomotor network as expected, including frontal eye field, for the target (left) hemisphere (where there is no lesion). No activation was found in the right hemisphere, possibly due to full control of the healthy hemisphere during the task. The activation time-course for a left ROI is shown in the bottom plot: the grey bars represent the time periods where activation should increase. Note the similar activation patterns between TMS conditions. (**C**) TMS Real: brain activation during the saccade task (stimuli > baseline) in both hemispheres. Increased activation was also found in the right (lesioned) hemisphere. The activation time-course reveals a significant effect of the stimulation for the right ROI, as depicted in the plot. This map reveals a positive effect of the stimulation (inhibition of the target hemisphere) that brings the lesioned (right) hemisphere ‘back to the game.’ Results are shown at *P* < 0.008 corrected at cluster level (cluster-defining threshold *P* < 0.05). Error bars in the time-course plots reflect the standard error of the mean (SEM). Random effects (RFX) Blood oxygenation level-dependent (BOLD) analysis (N = 13).

To investigate if the changes in neurotransmission, suggesting synaptic adjustment, were associated with changes in brain activity consistent with the imbalance hypothesis, we tested a simple fMRI saccade task. Specifically, we expected that TMS inhibitory stimulation to the healthy hemisphere should restore balance because of disinhibition of the contralateral lesioned hemisphere, leading to rebalance.^24,41^ We found that the target (healthy) hemisphere was significantly activated during the saccade task under both TMS conditions (GLM *t*-test: 3.20 < t(12) < 8.00; *P* < 0.008, d = 0.46). Additionally, a significant interaction between task and TMS condition was observed, supporting a TMS-induced effect in the left (healthy) IPS (Fig. 5A). Importantly, we found a 6-fold increase in activity in the lesioned (right) hemisphere following real ipsilateral inhibitory TMS, compared to a 1-fold change in the healthy hemisphere—suggesting contralateral disinhibition. This was confirmed by a random effects general linear model (GLM) analysis of the fMRI data (see activation maps for both sham and real TMS conditions; Fig. 5B and C). Significant activation was observed in the lesioned hemisphere, specifically after real TMS. Time-course data further illustrate a clear stimulation-dependent increase in right hemisphere activity, which was significant only after real TMS (t(12) > 3.20, *P* < 0.008, d = 2; Fig. 5C).

### TMS-induced reduction of Glx levels and GABA variance

**Figure 6:**
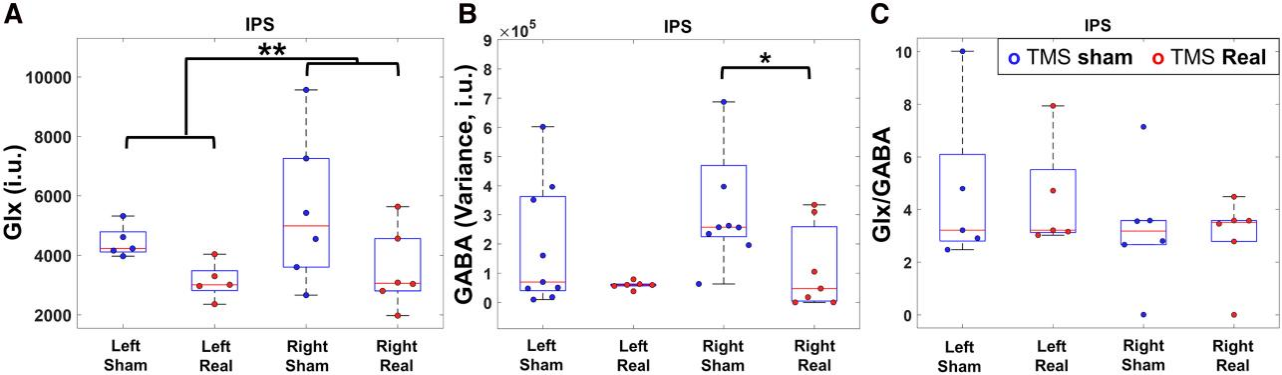
**MRS measures in IPS: impact on Glutamate levels and GABA neurotransmitter level variance.** (**A**) Glx study: we found reduced Glx with TMS stimulation (Glx TMS Sham versus Real (Left AND Right): F(22) = 8.90; *P* = 0.009; effect size *η*2 = 0.262; N = 13). (**B**) GABA quantification results. Data revealed a significant effect of the TMS protocol in variance reduction in the right (lesioned) hemisphere (contralateral to the stimulation) (Levene F(14) = 5.46, *P* = 0.035, d = 0.88). (**C**) Glx/GABA ratio show a tendency to a reduction in the lesioned hemisphere. Each individual data point represents a single patient.

The stimulation target region was the left IPS, and we performed ROI-based analyses (IPS left and right) of MRS GABA and Glx (Glutamatergic substances) data. MRS allowed detecting consistent neurochemical changes following the TMS. It is important to note that our quality-control metrics for MRS give high confidence in the metabolite quantification results. Average CRLB/SNR for the left ROI: GABA = 6.09 ± 3.63%/14.84 ± 4.75; Glx = 3.88 ± 2.47%/23.41 ± 8.07. Average CRLB/SNR for the right ROI: GABA = 5.61 ± 2.49/15.59 ± 3.67; Glx = 3.59 ± 1.81/25.55 ± 8.66. Accordingly, a planned group analysis of Glx levels (Fig. 6.A.) revealed a differential response between TMS real and TMS sham stimulation, with significantly reduced concentration of glutamatergic molecules (Glx) after real stimulation for both hemispheres (F(1,22) = 8.90; *P* = 0.009, d = 0.26). Strikingly, TMS also significantly reduced the GABA variance in the right hemisphere (Levene F(1,14) = 5.46, *P* = 0.035, d = 0.88; Fig. 6.B.), showing that reduction of postsynaptic variance (as identified in PET data) is also accompanied by a similar effect at the presynaptic level. This was further confirmed by a tendency to the reduction of Glx/GABA ratio (Left Sham: 4.67 ± 1.39 (Mean ± Standard error); Left Real: 4.40 ± 0.93; Right Sham: 3.29 ± 0.94; Right real: 2.97 ± 0.63).

### Correlated increase of GABA neurotransmitter and receptor levels, as revealed by PET and MRS, suggests synaptic plasticity

**Figure 7:**
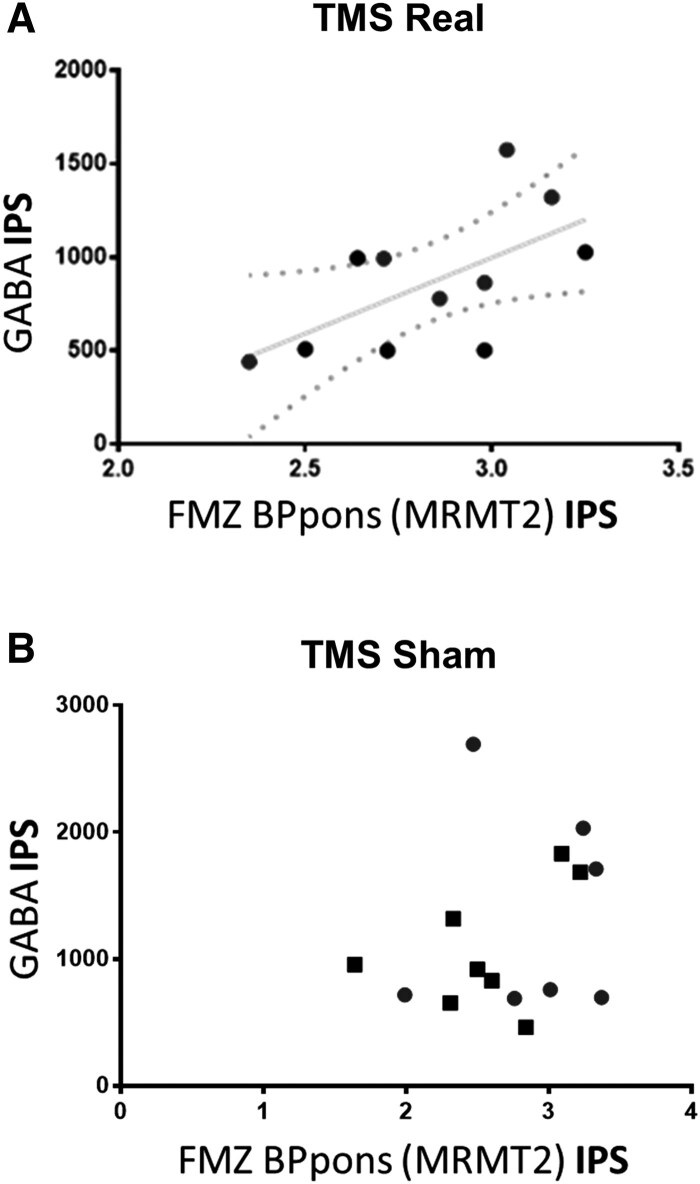
**Correlations of PET and MRS data showed association of pre- and post-synaptic measures suggestive of increased covariance across synaptic compartments.** Scatter plots of PET-FMZ (GABAa receptors) versus MRS (GABA) quantification in the IPS region of interest are shown for both TMS conditions (A- real; B-sham). Significant correlation of GABA receptor binding (PET) and GABA (MRS) levels is present only when TMS is Real (r(11) = 0.64, P (Spearman) = 0.037; N = 11). Each individual datapoint represents a single patient.

The association between pre- and post-synaptic effects was confirmed by the finding that GABA levels were associated with GABA_A_ receptor binding only when TMS is real (r(11) = 0.6424, P(Spearman) = 0.037). This effect mainly stems from the lesioned hemisphere when TMS is real (r(6) = 0.518, P(Spearman) = 0.048), suggesting a long-range effect of TMS on regulation of neurotransmission (Fig. 7).

The functional significance of GABA receptor levels was established at baseline by the observation that they were significantly associated with neuropsychological scores of parietal (left IPS) function in the absence of TMS (Landmark test, r(8) = 0.81, P = 0.015) and right parietal (IPS; Token test total score v r(7)=−0.777, *P* = 0.04).

In sum, real stimulation led to increased coupling between GABA neurotransmitter levels and its receptors, suggesting fine-tuning of inhibitory neurotransmission. This aligns with the hypothesis of modulation of the (im)balance between hemispheres, supporting the use of TMS as a potential treatment approach to disorders of hemispheric asymmetry. This modulation of hemispheric interactions was accompanied by changes consistent with synaptic sharpening (reflected by reduced variability of pre- and post-synaptic molecular markers of inhibition and reduced synaptic noise), a process thought to support neuroplastic stabilization.^22^

## Discussion

Our multimodal molecular imaging, spectroscopic and functional imaging data from the same day in stroke patients submitted to sham or real stimulation provide evidence for pre- and post-synaptic sharpening at inhibitory synapses, which is a mechanism of neuroplasticity^22^ consistent with a model of a statistically optimal excitation/inhibition balance through variance reduction.^8^ Our results provide a mechanism of action that has direct implications for treatment approaches in stroke relying on interhemispheric inhibition (IHI) using TMS. Our results suggest that modulating contralesional IPS promotes rebalancing of interhemispheric interactions via disinhibition. However, alternative models of poststroke cortical dynamics and network reorganization could equally explain some of the effects we observe.^24^ Carson (2020) has argued that some applications of the interhemispheric competition model—particularly those relying on TMS-derived M1 IHI—rest on misunderstandings of callosal physiology. Specifically, he emphasizes that interhemispheric projections do not exert ‘undifferentiated inhibition’ or produce simple excitability asymmetries; rather, they may support contrast-enhancing, integrative functions through mechanisms such as crossed surround inhibition and, in some contexts, crossed facilitation. These mechanisms narrow the excitatory focus in a highly differentiated, region-dependent manner, and Carson contends that the IHI technique may mask these properties because it interrogates the system in an artificial way. Importantly, however, Carson’s critique is directed at TMS-based inference in M1, whereas our study targets IPS, an association area with different callosal connectivity and functional roles. Our conclusions therefore do not rely on the assumption of undifferentiated interhemispheric inhibition in motor cortex nor on TMS-derived measures that Carson questions, but rather on molecular imaging and neurochemical features. Nevertheless, we acknowledge this ongoing debate, which calls for further comparative work.

It is important to note that PET and MRS assess distinct facets of GABAergic function: while PET primarily reflects synaptic receptor binding, MRS captures tissue GABA concentrations, which are more closely associated with synthesis in pre-synaptic compartments.^42^ This distinction has important implications for interpreting the neurobiological specificity of our findings. The precise cellular and functional sources of the MRS signal remain partially unresolved. Nevertheless, the large pool of presynaptic GABA is complementary to the dominantly postsynaptic GABA receptor pool from a compartmental point of view.

Our study generalizes to humans the notion that relevant synaptic parameters include variance and covariance in neural measures,^4^ which are relevant for synaptic plasticity.^5^ Our TMS protocol likely modulated the number of synaptic inputs, for which prior modelling work suggested that they depend on the variability of postsynaptic responses.^6^ We hypothesize that decreased variability of drive to interneurons in the nonlesioned hemisphere may help shape short and long-term plasticity. Our results support the interpretation of a recent postmortem and computational study in schizophrenia, which showed that increased variability of excitatory drive to fast-spiking parvalbumin interneurons leads to lower prefrontal gamma power,^7^ which is known to be important to promote plasticity.^43^ Importantly, GABA-A receptor changes may not be confined strictly to the IPS but rather distributed across adjacent parietal and non-parietal regions involved in interhemispheric rebalancing. As our PET results seem to point out, a whole-hemisphere measure may be more sensitive to network-level effects, while an anatomically constrained ROI might miss them. This supports the idea that TMS effects, particularly in the context of poststroke plasticity, may be better captured at a network or hemispheric level rather than a single region.

Optimization of synaptic coupling across hemispheres would favour plasticity through a statistically optimal E/I balance. Variability of pre/post changes due to optimizing postsynaptic response statistics (including enhanced covariance, as shown by our data) leads to a framework where the full distribution of postsynaptic responses (instead of merely the mean weight) is optimized through joint pre- and post-synaptic modifications that are governed by a set of tightly coordinated neurotransmitters. Previous studies provide distinct correlation patterns regarding GABA MRS and PET correlations in health and disease. While Koen^44^ show no significant associations in a healthy ageing study, Violante^45^ reported a negative correlation in a neurodevelopmental disorder of disrupted GABA balance, neurofibromatosis type 1. In addition, succinic semialdehyde dehydrogenase (SSADH) deficient patients show widespread reduction in receptor binding on [(11)C]-flumazenil-PET in association with high endogenous brain GABA levels.^46^ Why this negative correlation becomes more apparent in genetic disease states suggests that stronger pre- versus post-synaptic homeostatic interactions do occur in disease states, either genetic or acquired. It remains intriguing why the correlation identified here is positive, suggesting that it goes beyond a simple homeostatic mechanism, and probably reflects an initial plasticity mechanism (strong pre- and post-synaptic correlation, similarly to what is observed in physiological studies). This finding might serve as a starting point for further mechanistic investigations in neuromodulated clinical populations.

Furthermore, we found functional evidence that can be interpreted in light of the interhemispheric inhibition model and a mechanism of action for inhibitory TMS in the hemisphere contralateral to the lesion in stroke. The bilateral response to a unilateral TMS can be interpreted in several ways. We show that inhibitory TMS induced improved coupling between GABAergic pre- and post-synaptic function and enhanced contralateral activation, thereby restoring hemispheric balance in association with reduction of molecular signal-to-noise ratios. Moreover, we show a reduction in the excitatory glutamate/glutamine pool within the target (healthy) region, and recovery of neural responses in the contralateral (lesioned) regions in unilateral stroke. With this cTBS protocol, we observed an increase in visual activation following stimulation, much more pronounced at lesioned hemisphere, and a decrease in both GABA and glutamate. This pattern has already been reported elsewhere.^47,48^ The 6-fold contralateral increase in activation may reflect a rebalancing of interhemispheric dynamics. The observed decrease in both GABA and Glutamate concentrations likely reflects homeostatic mechanisms to preserve excitation inhibition balance. This is in line with recent findings, suggesting that this TMS protocol may sometimes produce paradoxical net facilitation at the network level.^19,20^ This empirically adds to the model of interhemispheric inhibition in vivo, coupled with pre- and post-synaptic tuning. Our data further reveal that noninvasive brain stimulation is able to recover hemispheric balance upon unilateral lesions, although our study being limited by the fact that precise localization of the site of stimulation (e.g. using MRI scan) was not performed. We tried to overcome this using a specific round coil to ensure that we have stimulated relevant parts of the parietal cortex in all patients. A more focal approach using a figure-of-eight coil would have run the risk of missing the target. Moreover, bilateral response to a unilateral TMS can vary, depending on the specific location of the stimulation, the intensity of the stimulation, and the characteristics of the individual being studied, and thus, this can be a limitation of our study. On the other hand, we included patients with a relatively large interval poststroke (1–8 weeks, albeit all prior to a chronic state), and this should be considered in future studies.^9^

A striking aspect of our data is the relatively long-lasting duration of neuronal and hemodynamic effects induced by a short TMS protocol: we applied TMS a few minutes before each PET scan and up to 2 h before MRI (with significant effects in the lesioned hemisphere). This extends the timeline suggested by studies using similar stimulation protocols^29,49^ and aligns with previous findings reporting that protocols of cortical inhibition in poststroke populations may extend duration beyond typical windows due to pathophysiological changes/brain reorganization after lesion^50,51^ that may reflect delayed hemodynamic/metabolic effects, also on GABA measured using MRS.

This ability of TMS to alter neurotransmission, as demonstrated here, suggests that transcranial brain stimulation can indeed be used to foster brain plasticity^29^ in a number of neurological and neuropsychiatric disorders.^41,52^ Variance reduction has indeed been shown to represent a robust neuroplasticity mechanism.^4-6^ The functional link between receptor availability and neurotransmitter balance following stimulation provides further evidence for synaptic plasticity based on coupling optimization. Uncovering determinants of these effects can only be achieved using a multimodal imaging approach.^53-55^

As for the principle of brain lateralization, using an innovative multimodal approach in a unique patient group, our data provide evidence of hemispheric asymmetry and its association with synaptic plasticity mechanisms, with potential impact on recovery mechanisms, paving the way for mechanism-driven clinical applications to treat disorders of interhemispheric imbalance, in particular in stroke.

## Data Availability

No new code was generated for data analysis in this study. Analyses were conducted using publicly available toolboxes (e.g. Gannet for MRS), and data are available from the corresponding author upon reasonable request.
